# ASO Author Reflections: Preoperative Biliary Drainage—A Controversial Intervention with a Complicated Past

**DOI:** 10.1245/s10434-026-19649-3

**Published:** 2026-04-14

**Authors:** James Y. W. Lau

**Affiliations:** Prince of Wales Hospital, The Chinese University of Hong Kong, Shatin, The People’s Republic of China

## Past

We present a multicenter, noninferiority trial comparing preoperative biliary drainage (PBD) using a self-expanding metal stent (SEMS) to early surgery without PBD in patients with planned curative-intent surgery to treat pancreatic or periampullary cancer who were not receiving neoadjuvant therapy.^1^

Over the past 20 years, the status of endoscopic PBD has shifted. Once an accepted intervention based on inconsistent evidence, it became a controversial option due to safety concerns. These concerns increased after van der Gaag et al’s 2010 randomized trial reported that routine preoperative biliary drainage using plastic stents in patients undergoing surgery for cancer of the pancreatic head increased the rate of complications.^2^ A 2016 extension of this trial concluded that fully covered SEMS yielded better outcomes than plastic stents.^3^ It concluded that early surgery without PBD remained the treatment of choice.^3^

Meanwhile, advances in chemotherapy led the National Comprehensive Cancer Network (NCCN) to recommend in 2020 that neoadjuvant treatment, rather than upfront surgery, should be first-line treatment for borderline resectable pancreatic cancer.^4^ The very definition of “borderline resectable” continued to evolve. By the time the lengthy enrollment period of our clinical trial ended (2013–2021), both surgical and oncologic “best practice” for pancreatic cancer treatment had changed markedly. The evolution complicates the interpretation of our trial results as contemporary clinical guidelines recommend against routine preoperative biliary drainage in patients not on neoadjuvant therapy. However, critical data remain scarce on the specific subpopulations for whom the benefits of PBD might outweigh the risks.

## Present

Although the state of evidence moved on, clinical practice has lagged behind. An analysis of Dutch national registry data on guideline compliance from 2012 to 2017 (14,491 patients, 2290 resections, 4561 with chemotherapy) found most quality indicators did not change over time. For instance, 62.7% of patients with a resectable head tumor and bilirubin > 250 μmol/L continued to receive preoperative biliary drainage—a practice at odds with prevailing guidelines.^5^

This persistent clinical gap is relevant to our trial. The serum bilirubin range of > 250 μmol/L exceeds the maximum eligible level in van der Gaag’s trial^2^ but was intentionally included in ours. For participants in the high-bilirubin subgroup who underwent curative intent resections (41 PBD, 39 early surgery), we found surgery-related complication rates of 17.1% for PBD versus 28.2% for early surgery.^1^ These data underscore the need to interpret our results in light of current practice, not just clinical practice guidelines. This is especially true for the use of PBD with SEMS in patients with the most severe obstructive jaundice, where practice patterns appear to be most entrenched.

## Future

Future studies should focus on maximizing patient comfort and safety throughout the care continuum, from cancer diagnosis to the transition to palliative care. Based on the current literature, we see the most significant evidence gap in patients with serum bilirubin > 250 μmol/L, who were systematically excluded from previous key trials.^2,3^ We encourage further studies focusing on the use of fully covered SEMS in this specific patient population. This is particularly critical given that multiple studies have found higher rates of adverse events with plastic stents in patients with obstructive jaundice. Future studies should be conducted at high-volume centers with experienced endoscopists and surgeons to avoid operator bias. Rather than marking the end of inquiry on the topic, we hope our clinical trial inspires thoughtful new study designs. The goal is to improve clinical decision-making by tailoring pancreatic cancer treatment regimens to each patient’s unique needs and risks.
